# TNF-α and Beyond: Rapid Mitochondrial Dysfunction Mediates TNF-α-Induced Neurotoxicity

**DOI:** 10.4172/2155-9899.1000467

**Published:** 2016-11-14

**Authors:** Ashley E. Russell, Danielle N. Doll, Saumyendra N. Sarkar, James W. Simpkins

**Affiliations:** Physiology & Pharmacology, Center for Basic and Translational Stroke Research, Blanchett Rockefeller Neuroscience Institute, West Virginia University, Morgantown, West Virginia, USA

**Keywords:** TNF-α, microRNA-34a, Oligomeric Aβ, Mitochondria, Electron transport proteins, Stroke, Alzheimer’s disease

## Abstract

This short communication describes our research which demonstrates that TNF-α causes a rapid decline in mitochondrial function, leading to neuronal cell death. As such, this neurotoxic proinflammatory cytokine may play a role in brain damage from stroke and neurodegeneration in chronic conditions such as Alzheimer’s disease (AD) and Parkinson’s disease. We have extended this initial observation by demonstrating that TNF-α stimulates a microRNA (miR-34a) which we have shown reduces five key proteins in the mitochondrial electron transport chain through base-pair complementarity. miR-34a is increased in affected brain regions of Alzheimer’s patients and transgenic AD mouse models. We have further shown that oligomeric amyloid beta 42 (oAβ42) stimulates miR-34a. Collectively, these data suggest that TNF-α, oAβ42, and miR-34a participate in a vicious cycle, resulting in mitochondrial dysfunction, which is critical to the neuropathology of AD.

## Short Communication

In the report by Doll et al. [1], we demonstrated that administration of pathophysiologically relevant doses of TNF-α to cultured cells leads to a rapid decline in mitochondrial function as expressed by decreases in basal respiration, ATP production, and maximal respiratory capacity. This effect was seen as early as 1.5 hours after exposure in both a hippocampal cell line (HT-22) and in primary neurons. This effect appears to be mediated through TNF-α receptor 1 (TNF-R1), subsequent increase in caspase 8 activity, and decline in mitochondrial membrane potential, which resulted in a release of cytochrome C from the mitochondria into the cytosol. Cytochrome C shuttles electrons between Complexes III and IV in the mitochondrial electron transport chain (ETC), and with its diminished availability, the ETC is no longer able to function properly and decreases in basal respiration, ATP production, and maximal respiratory capacity are observed.

TNF-α is a proinflammatory cytokine, known to be increased during inflammation [2] and can play a role in brain damage from stroke [3,4]. Doll et al. [1] indicate a potential mechanism by which TNF-α can exacerbate stroke damage: through a rapid and profound decline in neuronal mitochondrial function.

With this knowledge, we can speculate that mitochondrial dysfunction may play a role in brain damage associated with periods of neuroinflammation, including both acute brain damage from stroke and traumatic brain injury, as well as in chronic neurodegenerative conditions like Alzheimer’s disease (AD) and Parkinson’s disease. It is well established that neuroinflammation plays a role in AD, but whether it be a bystander or key perpetrator is still widely discussed within the scientific community. Post mortem brain analyses have shown elevated levels of proinflammatory cytokines, including TNF-α, in AD brains when compared to non-demented controls [5]. Chronic neuroinflammation observed in AD may be the result of activation of the innate immune system from oligomeric amyloid β 42 (oAβ42), [6], a key component for the formation of neuritic plaques.

Mitochondrial dysfunction and global reductions in energy metabolism have been implicated in AD for several decades [7,8]. Evidence suggests that energy deficits stemming from dysfunctional mitochondria may cause the amyloid precursor protein to be processed in a pathological, amyloidogenic pathway, resulting in the oligomerization of Aβ42 [9–11]. This increases inflammation and subsequent release of pro-inflammatory cytokines like TNF-α from neurons, microglia, and astrocytes [12]. Our laboratory has observed suppression of mitochondrial function in primary rat neuronal cultures after exposure to oAβ42 [13], and we speculate that this effect is mediated through induction of TNF-α.

TNF-α binding to TNF-R1 can activate the transcription factor NFκB. Our laboratory has recently observed an NFκB binding site on the promotor region of microRNA-34a (miR-34a). MicroRNAs (miRs) are small, non-coding RNAs that repress protein translation by base-pairing with mRNAs and inhibiting translation or targeting mRNA for degradation [14–16]. Precursor miRs created within the nucleus are transported to the cytoplasm for processing by DICER, resulting in mature miRs; these can either be loaded into the RNA-induced silencing complex (RISC) to exert their repressive effects on mRNAs within the parent cell, or be packaged into multi-vesicular bodies (MVBs) [17]. Sorting of miRs into MVBs may occur from the interaction of raft-like regions on MVB limiting membranes with specific miR motifs or RNA binding protein (RBP)-miR complexes [17,18]. These interactions result in the internalization of miRs as intraluminal vesicles (ILVs), which can be released from the cell as exosomes when the MVB fuses with the plasma membrane of the cell [17–19].

**Figure 1:**
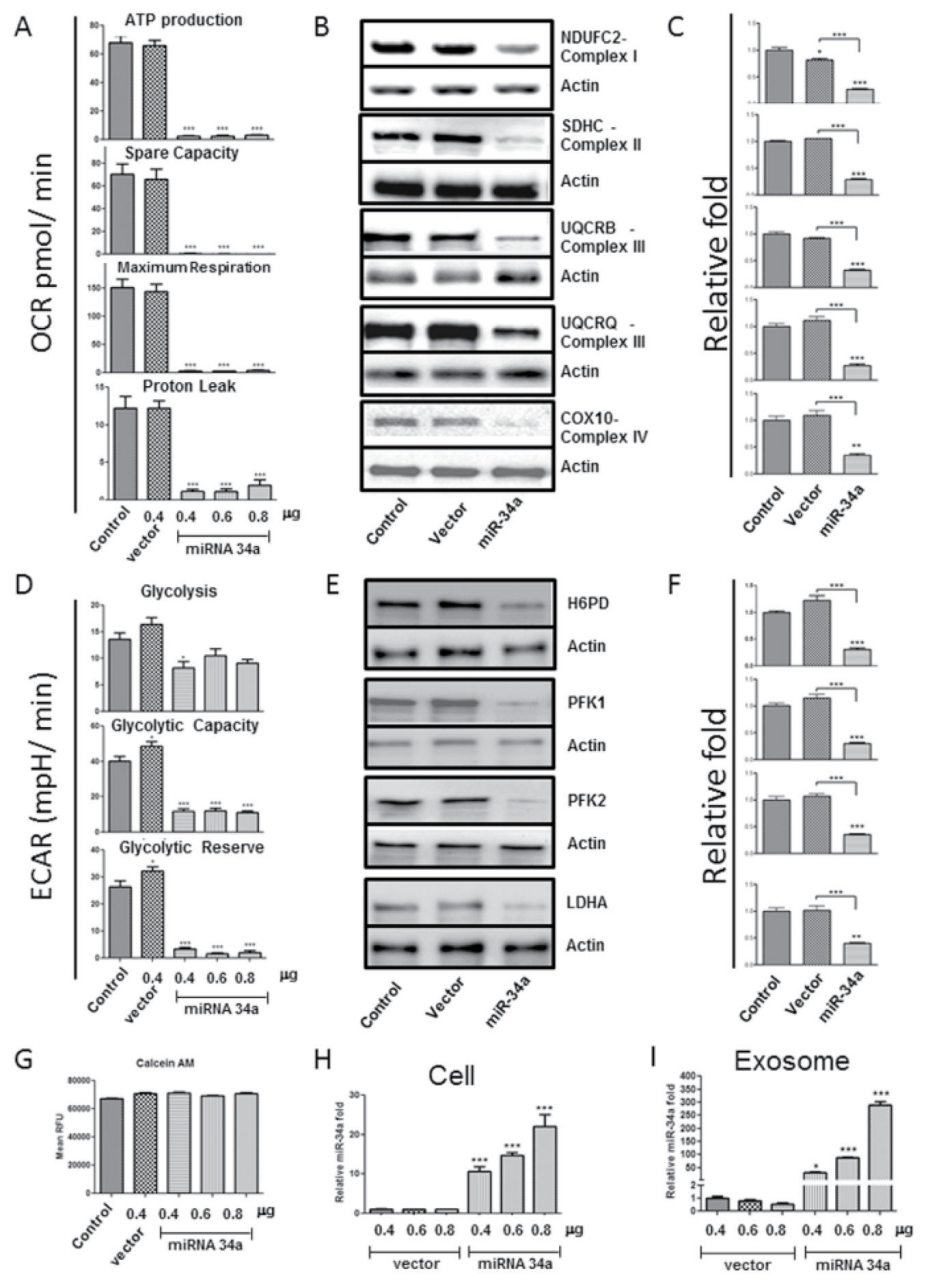
Functional effects of ectopically expressed miR34a on oxidative phosphorylation and glycolysis in primary neurons. (A) ATP synthesis, spare respiratory capacity, maximal respiration, and proton leak was measured in rat primary neurons (E18, 7DIV) 36 h after transfection with empty vector or with three increasing concentration of miR-34a-expression vector. (B) Representative western blot of oxidative phosphorylation proteins, NDUFC2, SDHC, UQCRB, UQCRQ, and COX10 probed with respective protein specific antibody in protein samples isolated from the transfected primary neurons. β-actin was used as normalization controls. (C) Densitometric quantification of respective protein levels are shown in the adjacent bar graph as fold difference, average of control =1, data are mean 7 ± SEM, n=3 independent transfection experiments. (D) Glycolysis rate was measured in rat primary neurons (E18, 7DIV) thirty six hour after transfection with empty vector and with three increasing concentrations of miR-34a-expression vector. (E) Representative western blots of enzymatic glycolysis proteins, H6PD, PFK1, PFK2, and LDHA probed with respective protein specific antibody in protein samples isolated from the transfected primary neurons. β-actin was used as normalization controls. (F) Densitometric quantification of respective protein levels are shown in the adjacent bar graph as fold difference, average of control =1, data are mean 7 ± SEM, n=3 independent transfection experiments. (G) Viability of the miR34a expression plasmid transfected neurons were determined by CalceinAM assay. Level of miR-34a in transfected neurons (H) and in exosomes isolated from the transfected cell culture medium (I) were determined by qRT-PCR. ^*^p 0.05, ^**^p 0.01 and ***p 0.001. Reprinted from: Sarkar S, Jun S, Rellick S, Quintana DD, Cavendish JZ, et al. (2016) Expression of microRNA-34a in Alzheimer’s disease brain targets genes linked to synaptic plasticity, energy metabolism, and resting state network activity. Brain Research 1646: 139–151. Copyright (2016), with permission from Elsevier.

Loading of miRs into exosomes appears to be dependent on levels of both miR and the target mRNAs within the parent cell, i.e. if a cell begins producing high levels of a specific miR and its intracellular target levels remain the same, there will be a higher amount of miR detected in secreted exosomes [17]. In conjunction with these data, recent work from our laboratory has demonstrated similar findings [20]. Primary neurons were transfected with three levels of a miR-34a construct and we observed a 10–20 fold increase of miR-34a intracellularly, however we observed a robust increase of 50–250 fold when measuring levels of miR-34a in secreted exosomes (Figure 1) [20]. Exosomes have been viewed as intercellular communicators for some time now, and there are several ways in which they can interact with recipient cells, with most resulting in endocytosis of the exosome [17,18], allowing its contents to exert their effects on the recipient cell.

Because of base-pair complementarity, one miR can bind to and regulate many mRNAs, and one mRNA can be regulated by many miRs [16]. As stated above, of particular interest to both our laboratory and AD research is miR-34a, as some of its targets include five key proteins in the mitochondrial ETC; from Complex I: NDUFC2, Complex II: SDHC, Complex III: UQCRB and UQCRQ, and Complex IV: COX10. If miR-34a is preventing translation of these proteins’ mRNA, replacement after normal protein turnover will not occur, leading to a collapse of the ETC and subsequent mitochondrial dysfunction. Sarkar et al. [20] transfected primary neurons with a miR-34a construct and subsequently observed reductions in the five nuclear encoded ETC proteins described above, as well as substantial mitochondrial dysfunction (Figure 1).

**Figure 2:**
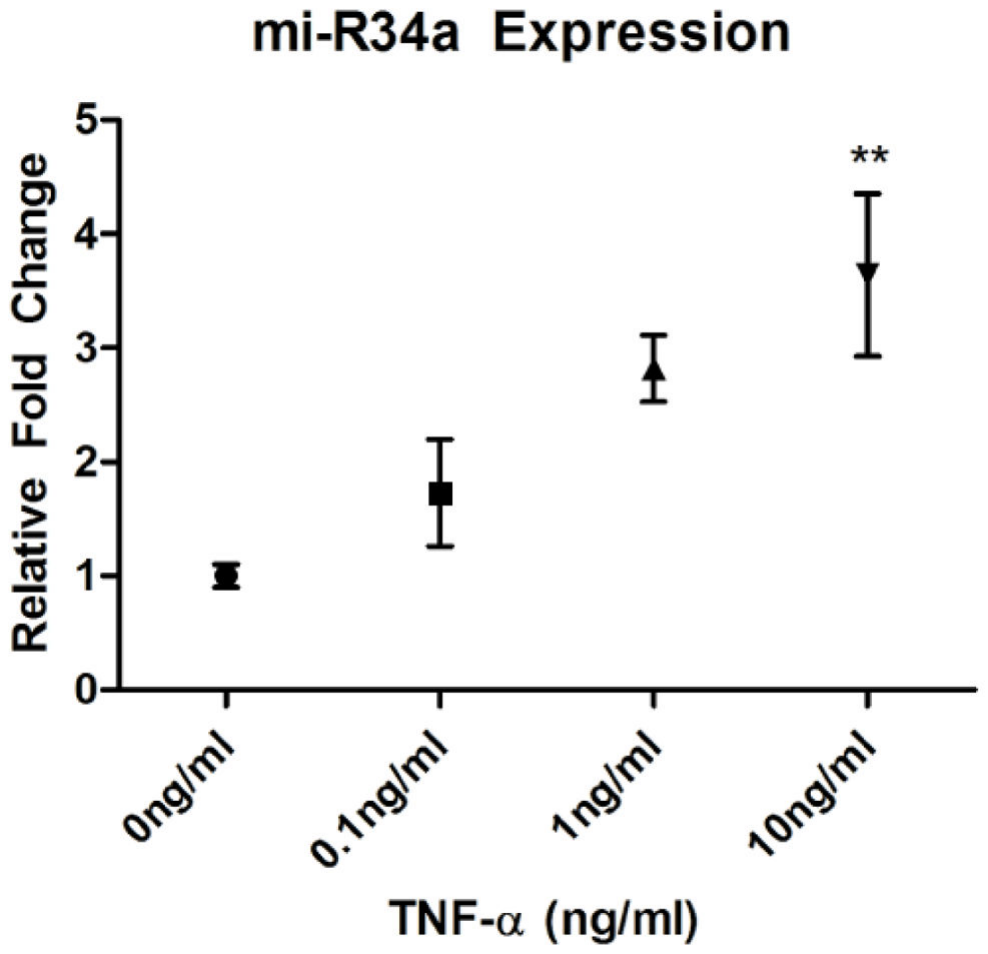
Scatter plot (± SEM) depicting the relative fold change of miR-34a levels in exosomes collected from media after a 24 hour exposure of 0, 0.1, 1, and 10 ng/ml of TNF-α in cultured HT-22 cells (^**^p<0.01). There is a relative increase in miR-34a content in secreted exosomes that is proportional to increased concentrations of TNF-α exposure.

**Figure 3:**
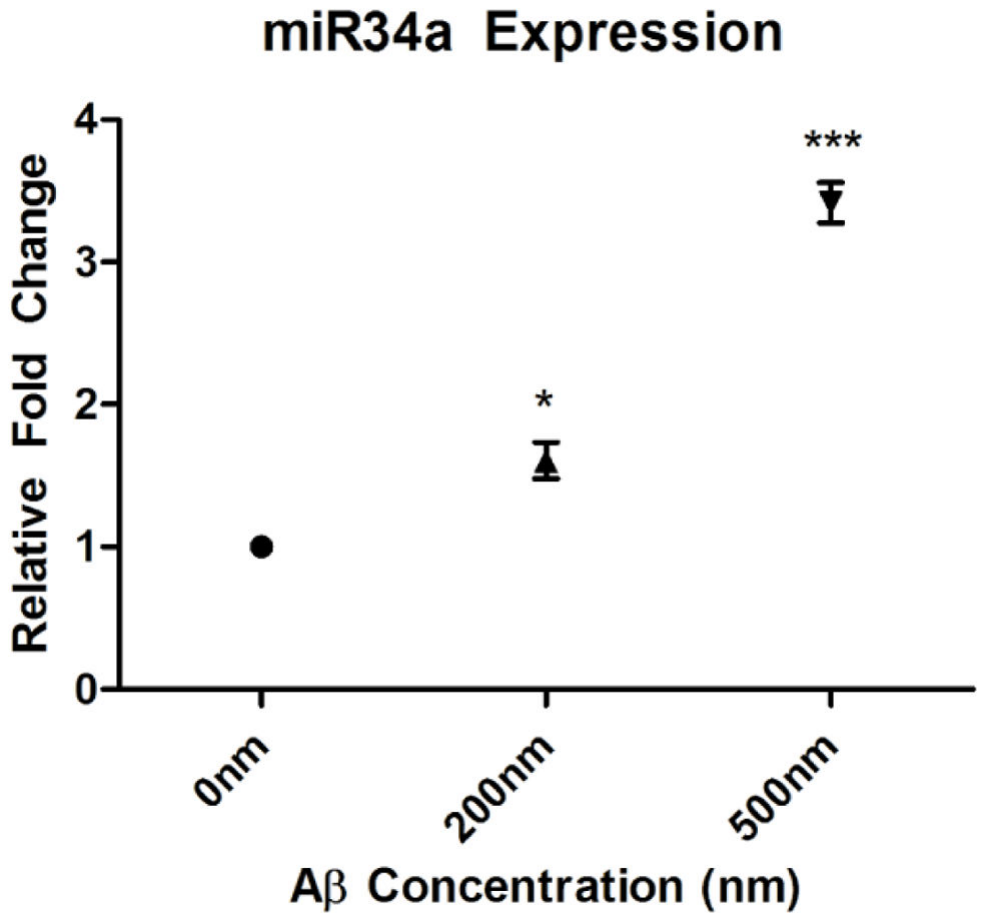
Scatter plot (± SEM) depicting the relative fold change of miR-34a levels in exosomes collected from media after a 24 hour exposure of 0, 200, and 500 nM oAβ42, in cultured HT-22 cells (^*^p<0.05, ^***^p<0.001). There is a relative increase in miR-34a content in secreted exosomes that is proportional to increased concentrations of oAβ42 exposure.

Preliminary unpublished data from our lab indicate that after 24 hours of exposure to TNF-α, there is a dose-dependent increase in miR-34a (Figure 2) as observed in exosomes collected from the media of exposed HT22 cells. In addition, we have preliminary evidence that oAβ42 exposure also leads to a dose-dependent upregulation of exosomal miR-34a after 24 hour exposure in HT22 cells (Figure 3). Our next step is to determine intracellular levels of miR-34a after exposure to these compounds, as well as determining whether or not the observed oAβ42-induced miR-34a upregulation is mediated through a pathway involving TNF-α.

**Figure 4:**
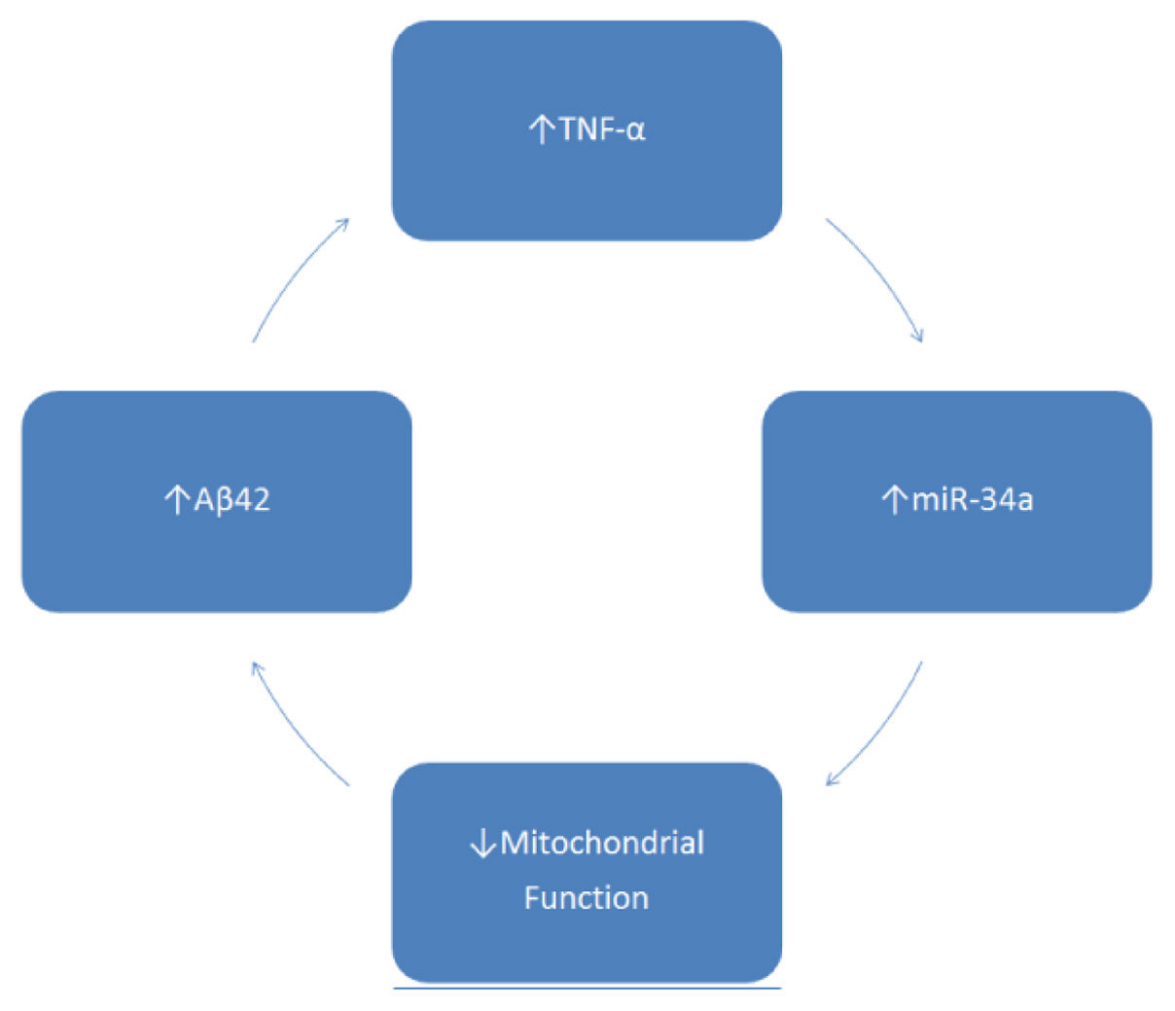
Proposed vicious cycle of AD progression. Mitochondrial dysfunction occurs in the AD brain, leading to increases in oAβ42. These aggregates lead to elevated inflammation, as seen by increases in levels of TNF-α, which leads to the upregulation of miR-34a. miR-34a inhibits translation of five key ETC proteins, preventing their replacement after protein turnover, and leads to a collapse of the ETC and therefore, mitochondrial dysfunction.

As discussed in the report by Doll et al. [1], TNF-α exposure leads to rapid mitochondrial dysfunction in both HT22 cells and primary neuronal cultures. Other work from our laboratory [13] has shown that exposure of primary rat hippocampal neuronal cultures to 200 nM oAβ42 induces mitochondrial dysfunction as well. We believe these effects are mediated through the pathways depicted in Figure 4, in that oAβ42 leads to increased release of TNF-α, which causes an upregulation of miR-34a that then leads to mitochondrial dysfunction, both within the parent cell and exosomal-recipient cells. Because exosomes can mediate cell-cell communication, miR-34a-containing exosomes secreted from a parent cell should theoretically be endocytosed by a neighboring recipient cell and exert its energy-impairing effects there as well. Once energy metabolism has been disrupted in this cell, it is speculated that there will be an increase in amyloid precursor protein processing towards a more amyloidogenic pathway [9–11], resulting in increasing amounts of oAβ42. In line with our hypothesis, we believe that increasing oAβ42 will increase inflammation, leading to the production of TNF-α, resulting in increased miR-34a and therefore miR-34a-containing exosome secretion. This vicious cycle will continue spreading throughout the AD brain, leading to neuronal cell death and eventually, observable symptoms in the patient.

Future work in our laboratory will focus on establishing these relationships and determining the role of this proposed vicious cycle in AD progression. We believe that by targeting certain aspects within the cycle therapeutically, we may be able to slow or even halt disease progression.
